# Navigating false positive HIV test results: a case report

**DOI:** 10.1128/asmcr.00097-24

**Published:** 2025-03-25

**Authors:** Q. Kaewpoowat, L. Stulken, M. D. Krasowski, B. Ford, J. T. Stapleton

**Affiliations:** 1 Department of Internal Medicine, Carver College of Medicine, University of Iowa 194453 , Iowa City, Iowa, USA; 2 Department of Pathology, Carver College of Medicine, The University of Iowa 160416 , Iowa City, Iowa, USA; 3 Iowa City Veterans Administration Medical Center, Iowa City, Iowa, USA; Pattern Bioscience, Austin, Texas, USA

**Keywords:** HIV-1, pregnancy, diagnostic algorithm, false positive

## Abstract

**Background:**

All Food and Drug Administration-approved laboratory-based human immunodeficiency virus (HIV) screening tests have high sensitivity and specificity. There is an HIV diagnostic test algorithm recommended by the Centers for Disease Control and Prevention (CDC) and Association of Public Health Laboratories (APHL) in the United States. Still, diagnostic challenges can occur in real-world practice. We describe an unusual case that exhibited false positive results for HIV screening and confirmation testing.

**Case Summary:**

A 27-year-old cisgender female had HIV testing as part of antenatal care. Her local HIV-1/2 antigen/antibody (Ag/Ab) screening test was reactive, and the HIV Ab differentiation assay was positive for HIV-1. Using CDC and APHL algorithm guidelines, she was diagnosed with HIV-1 infection. However, her HIV-1 ribonucleic acid (RNA) quantitative polymerase chain reaction was undetected. Although she immigrated from an HIV-1 high prevalence region, she was otherwise at low risk for HIV transmission. Qualitative HIV-1 deoxyribonucleic acid/RNA was undetected, and HIV-1 Western blot analysis was only positive for HIV-1 gp160 reactivity, confirming that her initial testing represented a false positive and that she did not have HIV infection. The rest of her pregnancy and delivery course was uneventful.

**Conclusion:**

Our case highlights the limitations of current HIV diagnostic tests and challenges in interpretation. Clinicians should be aware that false-positive tests are rare and be reminded to consider the pre-test probability when interpreting results.

## INTRODUCTION

Human immunodeficiency virus (HIV) screening tests have evolved over time. Newer tests detect early virologic and immunologic markers, shortening window periods (1). The Centers for Disease Control and Prevention (CDC) and Association of Public Health Laboratories (APHL) have updated the Laboratory Testing for the Diagnosis of HIV Infection accordingly (2, 3). Since 2017, screening with a fourth-generation HIV test (HIV-1/2 antigen/antibody [Ag/Ab] test) has been recommended as the initial testing method. All Food and Drug Administration (FDA)-approved laboratory-based HIV screening tests have high sensitivity and specificity for diagnosing established HIV infection (4, 5). Reactive HIV screening tests must be confirmed using an HIV-1/2 antibody differentiation test; discordant results between a reactive HIV-1/2 Ag/Ab screening test and HIV-1/2 antibody differentiation assay are reported as indeterminate and are resolved with HIV nucleic acid testing (NAT). Because the window period for NAT testing is the shortest, in the absence of antiviral suppression or elite controller status, HIV NAT testing is generally definitive (3). If HIV is undetected by NAT, a reactive HIV-1/2 Ag/Ab screening test with a negative HIV-1/2 antibody differentiation assay is classified as false positive (3). Alternatively, a concordant result reactive for both HIV-1/2 Ag/Ab screening test and HIV-1/2 antibody differentiation assay is considered sufficient to establish an HIV diagnosis regardless of HIV NAT results (3).

Despite the high sensitivity and specificity of the current HIV diagnostic algorithm, we describe a case with an unusual falsely reactive HIV-1/2 Ag/Ab screening test and HIV-1/2 antibody differentiation assay.

## CASE PRESENTATION

**Fig 1:**
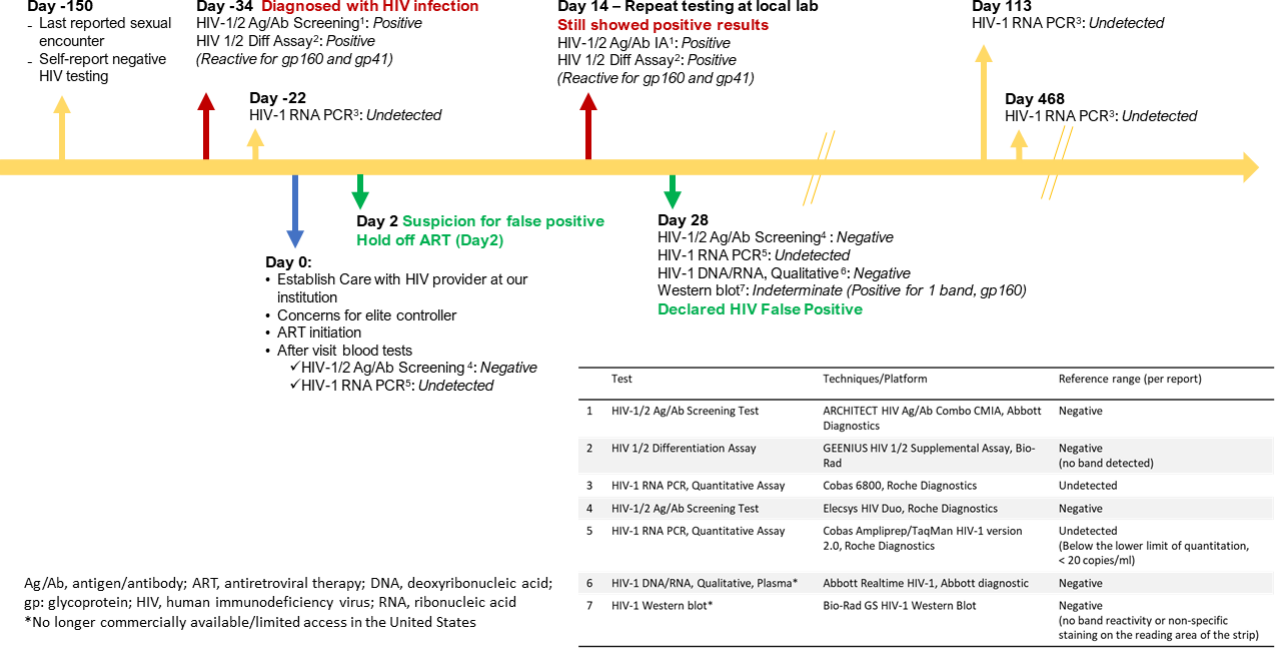
Timeline of the patient’s HIV-1 diagnostic tests.

A 27-year-old gravida 2, para 1 pregnant cisgender female with no significant past medical history, migrating from an HIV-1 high-prevalence region, presented to a local obstetrics clinic at 16 weeks gestation to establish antenatal care. At that time, a fourth-generation HIV-1/2 Ag/Ab screening test (ARCHITECT HIV Ag/Ab Combo, Abbott Diagnostics) was performed at a commercial reference laboratory (Mayo Clinic Laboratories, Rochester, MN) and was reactive (Fig. 1). An HIV-1/2 antibody differentiation assay (Geenius HIV 1/2 Supplemental Assay, Bio-Rad Laboratories) was performed at the same laboratory and was positive for HIV-1 antibodies against two HIV-1 proteins (glycoprotein [gp]160 and gp41) but negative for HIV-1 antibodies against p36, gp160, p24, and gp41. Further, HIV-2 antibodies (gp36 and gp140) were undetected. Her HIV-1 diagnosis was established based on current CDC and APHL recommendations that the presence of antibody recognizing two HIV-1 proteins represents a positive test. Her subsequent HIV-1 RNA quantitative polymerase chain reaction (PCR) (Cobas 6800, Roche Diagnostics) was undetected. Her absolute CD4 was 746 cells/mL (48%; reference range: 34%–62%).

The patient was referred to our tertiary care center for high-risk pregnancy care and HIV management. She was in disbelief of the HIV diagnosis. Since her last negative HIV test (conducted 3 months prior to presentation), she reported only monogamous sexual activity with a long-term male partner with unknown HIV status. She was separated from her partner, and his HIV status was unknown. She denied other risk behaviors for HIV acquisition and had no history of blood transfusion. She also denied HIV pre-exposure prophylaxis (PrEP) or experimental HIV vaccine exposure. Elite controller status, which presents as positive antibody in the face of negative HIV NAT confirmation, could not be ruled out. Therefore, antiretroviral therapy (ART), emtricitabine/tenofovir 200/300 mg and dolutegravir 50 mg per oral daily, was recommended at this first visit to prevent vertical transmission while working on additional evaluation.

We repeated HIV testing at our institution using a different HIV-1/2 Ag/Ab screening test (Elecsys HIV combi PT, Roche Diagnostics), and the result was negative. We repeated the HIV-1 RNA quantitative PCR (Cobas AmpliPrep/TaqMan HIV-1 version 2.0, Roche Diagnostics) at our institution (blood drawn prior to antiretroviral therapy), and it was undetected. After discussion regarding a concern for a false-positive initial HIV test with the patient, she elected to stop ART. The patient was exposed to ART for 2 days. To exclude a chance of discrepancy resulting from technical issues (e.g., specimen mix-up, mislabeling, improper handling), a repeat HIV test at her local hospital was requested. The HIV screening and confirmation tests were run with the same testing method as the original reference laboratory testing (Mayo) and showed identical results to those obtained previously (positive HIV-1/2 Ag/Ab screening test on the ARCHITECT HIV Ag/Ab Combo, Abbott Diagnostics; two of six HIV-1 antibodies positive on the Geenius HIV 1/2 Supplemental Assay, Bio-Rad Laboratories).

To address these discrepancies, repeat HIV-1 RNA quantitative PCR, qualitative HIV-1 DNA/RNA PCR, and HIV-1 Western blot (WB) were obtained. HIV-1 RNA quantitative PCR (Cobas AmpliPrep/TaqMan HIV-1 version 2.0, Roche Diagnostics) was still undetected, and HIV-1 qualitative DNA/RNA PCR (Abbott Realtime HIV-1, Abbott diagnostic) was negative. The HIV-1 WB (Bio-Rad GS HIV-1 Western Blot) was only positive for one band (gp160) and negative for eight others (p18, p24, p31, p40, gp41, p51/56, p65, and gp120), with an interpretation of indeterminate (6). The presence of two major viral bands is required for a positive interpretation (6). Upon review of all her test results, we concluded that she had false-positive HIV screening, and confirmatory test results and that she did not have HIV infection (Fig. 1). After she was referred back to her local obstetrician, repeat HIV-1 RNA quantitative PCR (Cobas 6800, Roche Diagnostics) was obtained during the third trimester, and again, HIV-1 RNA was undetected. The rest of her pregnancy and delivery course was uneventful. Repeat HIV-1 RNA quantitative PCR (Cobas 6800, Roche Diagnostics) at 1-year post-partum was reported as undetected, consistent with her HIV status being negative.

## DISCUSSION

HIV testing is a critical diagnostic procedure that clinicians must be proficient in interpreting. Despite the comprehensive guidance from CDC and APHL (3), interpretive challenges can occur in real-world practice. False-positive HIV testing usually refers to a situation when HIV-1/2 Ag/Ab screening is reactive, but a subsequent HIV-1/2 antibody differentiation assay is negative (not reactive), and HIV NAT is undetected. False-positive screening tests can occur due to technical issues (e.g., specimen mix-up or mislabeling, improper handling, and misinterpretation of a visually read point of care test) or biological causes (e.g., exposure to experimental HIV vaccine, allo- or auto-immune cross-reactivity, and some viral infections or medical conditions) (4, 5). False-positive HIV testing has been well described in some populations (7). Pregnant women have been known to have higher rates of false positives, likely associated with alloantibodies that cross-react with HIV during the antenatal period. In a study from Dallas, Texas, Adhikari et al*.* described a rate of false positives up to 17% in pregnant women using a fourth-generation test (8). In this study, the false positive was “confirmed” in the confirmation testing algorithm (HIV-1/2 antibody differentiation) but not using a different testing methodology. Our case is unique in that both the Architect HIV-1/2 Ag/Ab screen and Geenius HIV-1/2 antibody differentiation assays were reproducibly falsely positive from multiple patient specimens, while testing using alternative serologic assays and NAT was negative.

Since 2023, the CDC and APHL have updated their algorithm to allow for approved HIV-NAT testing to be used as a confirmation test (3). However, the main concept remains the same. HIV diagnosis is established if there is a concordant result between the HIV-1/2 Ag/Ab screening test and the HIV-1/2 antibody differentiation assay. Potentially, 1% of people living with HIV could spontaneously suppress HIV RNA in blood, so-called “elite controllers” (9). This population develops an appropriate immunologic response, resulting in positive HIV antibody testing but may have undetectable serum HIV-1 RNA viral load without antiviral treatment. While the high reliability of the current diagnostic algorithm is reassuring, it also underscores the recognition that this protocol is not without limitations.

First, although all fourth-generation HIV-1/2 Ag/Ab screening tests have excellent sensitivity and specificity, discordant results may still occasionally occur. Our patient had discordant test results between two fourth-generation HIV-1/2 Ag/Ab screening tests (ARCHITECT CMIA, Abbott Diagnostics; and Elecsys HIV combi PT, Roche Diagnostics). In a study by Krasowski et al. (10) comparing the performance between these two tests, there was not a statistically significant difference between methods (10). To enhance the performance of these HIV-1/2 Ag/Ab screening tests, a chemiluminescent signal or signal to cut-off ratio has been explored as a modality to predict HIV-1 infection confirmation (7, 8, 11). However, there is no standard cutoff point, and this number is not usually reported.

Second, understanding the structure of the human immunodeficiency virus and the principles underlying confirmatory testing is essential for test interpretation. Our patient had a positive HIV-1/2 antibody differentiation assay notable for antibodies to gp41 and gp160. Although this finding met the diagnostic criteria to confirm the presence of two HIV-1 antibodies, it raised suspicion when her quantitative HIV-1 serum RNA PCR testing was undetected, and her CD4 count was normal. The HIV-1 envelope protein gp160 is the precursor protein from which gp41 is cleaved (12). Following cleavage, gp41 and gp120 form envelope trimers that are responsible for binding to CD4 (12). Antibodies to gp41 would therefore also react with the precursor gp160. HIV-1 infection with these antibodies detected, but without antibodies to another non-enveloped HIV-1 protein (e.g., GAG or POL) is unusual, and these findings would not have met the criteria for a positive HIV-1 WB test (6). Although rare, it could occur in advanced-stage HIV, acquired immunodeficiency syndrome (AIDS), patients who had lost antibodies to other HIV-1 structural proteins. This situation was not applicable in this case as the patient did not have any signs and symptoms of AIDS, and her CD4 count was normal. Because of these results, we suspected that the patient had false-positive HIV testing. We used HIV-1 WB, which is no longer used in the HIV diagnostic pathway, to assess the presence of HIV-1 antibodies. True-positive HIV-1 infection would be expected to demonstrate a wide array of HIV-1 antibodies to different HIV-1 antigens in WB assays, especially if the infection lasts more than 60 days as in this case.

Another confirmatory parameter is detection of HIV-1 proviral DNA. This can be detected even in long-term progressors and elite controllers (13) and in those with HIV-1 infection whose plasma viral RNA is reduced to undetectable levels due to treatment with ART (14). In the case presented here, this test was not pursued. A negative HIV-1 DNA/RNA qualitative PCR test and an HIV-1 WB demonstrating only common gp160 cross-reactivity led us to conclude that she had a false positive test. This fundamental concept and approach were applied in other case reports. Joshi et al. (13) reported an interesting case of an elite controller pregnant female with HIV diagnosis established by positive HIV-1 WB and whole-blood HIV-1 proviral DNA qualitative testing. Hastie et al. published a similar case describing false-positive testing in a low-risk pregnant patient. HIV-1 proviral DNA was again negative (15). Ochola et al. reported a false-positive case in which gp41 and gp160 were positive in the HIV-1/2 antibody differentiation assay. As mentioned above, this scenario should raise a suspicion for a false positive (16).

Finally, HIV-1 proviral DNA and HIV-1 WB testing have been used to resolve conflicting HIV results in HIV-1 treatment-experienced patients (17) and those patients who are exposed to ART and PrEP (18). Some clinicians propose to incorporate HIV-1 proviral DNA into the societal HIV diagnostic guidelines (15). We are supportive of this comment with the caveats that results must be interpreted cautiously in the context of clinical and supplemental test status. There are neither FDA-approved tests for this purpose nor commercially available tests with well-characterized sensitivity and specificity in common diagnostic dilemmas. HIV-1 proviral DNA testing can also provide false-negative results and requires repeat testing (16, 17). Moreover, the test is not readily accessible. Currently, there is no FDA-approved HIV-1 DNA assay available in the United States (19). The options for NAT testing to detect HIV-1 proviral DNA are not widely available and infrequently used. In-house laboratory testing is impossible to validate and maintain for most laboratories. Further, HIV-1 WB was also discontinued from most reference laboratories. This may cause future challenges in resolving HIV diagnostic dilemmas.

In summary, our case highlights the limitations of the current HIV diagnostic tests and testing algorithm. Clinicians should be aware that although false-positive tests are rare, positive predictive value is usually unintuitively low in low-risk populations, particularly in pregnant women. There is a need to develop a standardized commercial HIV-1 proviral DNA assay and maintain the HIV-1 WB at reference laboratories, to be used when HIV diagnostic tests have discrepant results.
